# Agroecology and beyond: enhancing ecosystem services provided by natural vegetation and inventing “service weeds”

**DOI:** 10.3389/fpls.2024.1436310

**Published:** 2024-06-28

**Authors:** Ioannis Gazoulis, Panagiotis Kanatas, Stavros Zannopoulos, Metaxia Kokkini, Vasiliki Kontogeorgou, Nikolaos Antonopoulos, Ilias Travlos

**Affiliations:** ^1^ Laboratory of Agronomy, Department of Crop Science, Agricultural University of Athens, Athens, Greece; ^2^ Department of Crop Science, University of Patras, Mesolonghi, Greece; ^3^ Koniareio Citrus Institute, Ministry of Rural Development & Food, Kechries, Greece

**Keywords:** ecosystem services, weeds, natural vegetation, service weeds, agroecology

## 1 Introduction

Weeds are considered serious and troublesome pests for crops, and their management had always been at the core of plant protection (Oerke, 2006; Travlos et al., 2021). Despite the negative connotation of the term “weed,” referring to plants that are spatially and temporally unwanted, weeds can sometimes have some positive impacts in agroecosystems due to their utility as food, feed, and their multiple ecosystem services (Blaix et al., 2018; Gaba et al., 2020). Numerous field studies have shown that biodiversity is a key factor in determining ecosystem services (Cardinale et al., 2012; Tilman et al., 2014; Visconti et al., 2018). Consequently, balancing biodiversity and productivity in agroecosystems is a challenging goal that can provide additional benefits due to multifunctionality (Mitchell et al., 2014). The aim of this opinion article is to present the ecosystem services potentially provided by (or related to) weeds in agroecosystems and discuss their quantification and further enhancement in an agroecological context. The keystone question is whether and how weeds could be managed and turned into “service weeds,” similar to service crops, to contribute valuable ecosystem services.

## 2 “Service weeds” and how to invent and promote them

### 2.1 Ecosystem services potentially provided by the weeds and their assessment

Service crops, like cover crops, are cultivated in agroecosystems to provide non-marketed ecosystem services, diverging from food, fiber, and fuel production (Garcia et al., 2018). In this context, we suggest the term “service weeds” to describe spontaneous plants that can provide important ecosystem services with minimal disservices, under specific pedoclimatic conditions, in various agroecosystems, as parts of wider communities and with proper management. The need for the introduction of this term comes from the necessity to highlight that weeds are not always or universally unwanted and aims to shift the paradigm to exploit weeds by means of their management, and to understand biodiversity as part of the field and the agroecosystem.

Under specific conditions, weeds are associated with several disservices, like competition with crops for water, light, and nutrients, a host of harmful pests and diseases, significant yield reduction, an increase in frost risk and damage, fungal diseases, and poisonous effects on livestock. On the contrary, weed communities can contribute to agroecosystem services, among others, by providing food for different organisms; as hosts for pollinators and natural enemies; for soil stability, nutrient cycling, and improved water infiltration and facilitation of water acquisition by hydraulic lift, runoff, and erosion prevention (Dale and Polasky, 2007; Power, 2010; Petit et al., 2011). It is notable how weeds can reduce the sensitivity of several crops to pests, either by strengthening their defense mechanisms or by making them less attractive to the pests (Capinera, 2005; Fagundez, 2014; Blaix et al., 2018). The “push-pull” strategies, which involve the diversification of tolerant and trap crops, respectively, could also be extended to natural vegetation and thus mitigate the infestation of the crops (Shelton and Badenes-Perez, 2006). Weeds also reduce N leaching due to erosion mitigation and increased N uptake and fixation and enrich soil with carbon, other nutrients, and organic matter (Moreau et al., 2020). Rahman et al. (2009) found that floor vegetation consisting of naturally occurring weed species increased beneficial nematodes and organic matter near the soil surface, while the presence of various weeds is related to increased nutrient use efficiency of the crops, increased carbon storage, improved soil functionality, and conservation of biodiversity and wildlife (Salomé et al., 2016; Garcia et al., 2018). Thus, this subset of ecosystem services that is defined for agroecosystems will further support pollinators as an already defined indicator (Balvanera et al., 2014). Furthermore, according to several qualitative and quantitative modeling studies, climate change is likely to lead to pollinator decline, soil erosion, and other negative impacts, and therefore the importance of the contributions of nature to people and quality of life, i.e., ecosystem services, gets bigger (Martín-López et al., 2018).

In general, species diversification in space and time is used to provide and enhance ecological processes that support multiple ecosystem services (Garcia et al., 2018; Nijmeijer et al., 2019). However, biodiversity does not result in better ecosystem services *per se* since pedoclimatic and socioeconomic conditions also play a role, and the invaluable functional diversity is not always closely correlated with genetic diversity (Isbell et al., 2011; Mace et al., 2012; Balvanera et al., 2014; Gaba et al., 2015). In all cases, a scientific consensus has been reached that biodiversity loss reduces the efficiency of several provisioning ecosystem services and, hence, the contributions of nature to human wellbeing (Cardinale et al., 2012; Balvanera et al., 2014; Visconti et al., 2018). Therefore, the need for enhancement, assessment, and quantification of the several ecosystem services is imperative. While there are several well-known ecosystem services provided by specific weed species (e.g., nitrogen enrichment due to Fabaceae species), what is missing is the systematic and standardized promotion of ecosystem services in a validated and broadly accepted way for the agroecosystems and the upscaling from the single species to the communities’ level. Quantification of ecosystem services is the key to the development of the most suitable agroecosystems and the enhancement of ecosystem multifunctionality (Kremen and Ostfeld, 2005). Robinson et al. (2022) suggested the use of a large precision yield dataset to answer questions about how landscapes influence yield. Brooker et al. (2023) suggested that a focus on functional traits like height, rooting depth, and production of allelochemicals and associated trait complementarity will improve understanding of why combinations of cultivars, crop species, or crops and weeds seem to be more productive or resilient to climate, pests, and disease. According to them, a diversity of non-crop plants can provide resources necessary for in-field functional processes, both below and above ground (e.g., carbon input and resource continuity for pollinators and natural enemies). Furthermore, there are important interactions between ecosystem services, with these relationships being either synergistic or antagonistic (Gaba et al., 2015). This is valid for the ecosystem services potentially provided by the weed communities, with their presence often associated with increased organic matter, while at the same time, crop production could be hindered in the short term.

Comprehensive systems, methods, and approaches are needed to quantify the different ecosystem services with due consideration of the difference in the distribution of the ecosystem services provided (Johnson et al., 2010). For instance, the ecosystem services functional spatial unit (ESSU) concept proposed by Rafflegeau et al. (2023) could be a great tool and a sound base to build a protocol for ecosystem services quantification, modeling, and enhancement.

### 2.2 Enhancement of ecosystem services

The above-mentioned quantification of ecosystem services is not merely theoretical but rather necessary for the enhancement of these services through the identification, evaluation, and protection of weed communities that can be exploited for multiple purposes.

**Figure 1:**
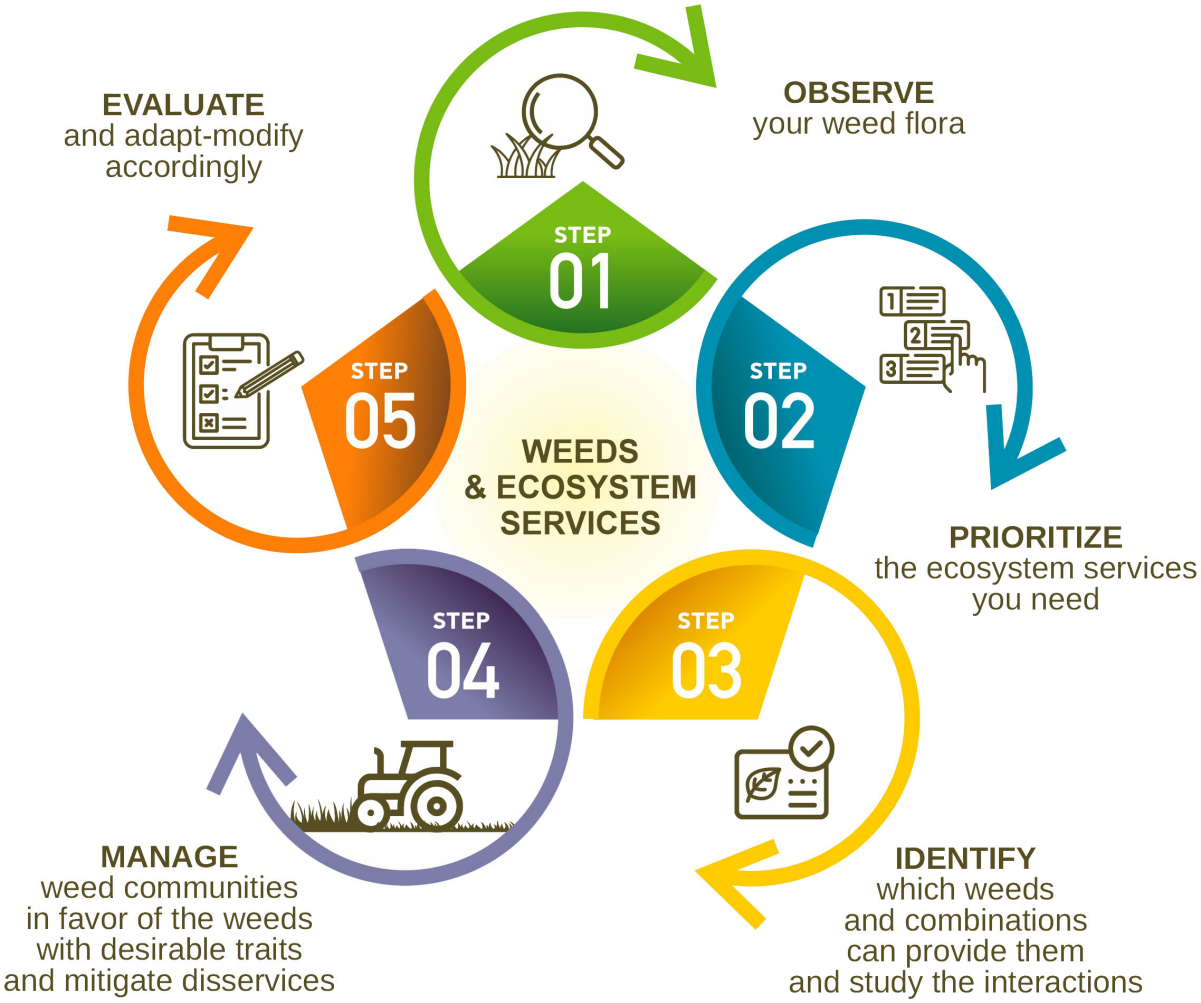
Steps to be taken in the framework of identification, assessment, and promotion of service weeds and service natural vegetation in agroecosystems.

We hereby propose a specific sequence of steps to be taken in the identification, assessment, and promotion of service weeds and natural vegetation in agroecosystems that can provide ecosystem services (**Figure 1**).

In our opinion, the good knowledge of the biology and ecology of weeds and the observation of the progressive differentiation of weed flora, both within fields and along field margins and boundaries, are very important for transforming them into “service weeds” (Step 1). Field boundaries and spaces between tree rows covered by service weeds, acting as “cover weeds,” that can contribute to important ecosystem services without risking crop health and productivity would be revolutionary. Field margins, fallow land, strips, and grasslands can increase biodiversity, promote diversification, and provide various ecosystem services to crops by creating better abiotic conditions for crop growth and productivity (Concepcion et al., 2012; Roschewitz et al., 2005; Gaba et al., 2015; Robinson et al., 2022). However, monitoring the composition of weed flora and its changes is the baseline, as these reflect specific conditions, agronomic practices, and the entire field history.

The next step (Step 2) involves the prioritization of several provisioning and regulating ecosystem services that add value to the specific agroecosystem and, in the medium and long term, would be beneficial for the farmers as well, exactly as proposed by Gaba et al. (2015) for crops. For instance, in poor soil with low fertility, the increase of soil nitrogen and soil organic matter would be of top priority, while in a sloppy orchard, the prevention of soil erosion and a good soil structure would be very important. Prioritization should be done in collaboration with farmers and other stakeholders. In this way, it will reveal their needs, raise their awareness and commitment, and help demonstrate good examples and success stories.

Step 3 refers to the identification of specific weeds and weed communities whose traits and complementarity can provide the most desired ecosystem services and consider their interaction and impact on the ecosystem. Indeed, each species is characterized by several functional traits linked to agroecosystem services (Barberi et al., 2018; Ciaccia et al., 2020). For example, depending on the priorities and the specific pedoclimatic conditions and weeds’ functional traits, we can either promote Fabaceae species for nitrogen enrichment or Poaceae species for structure improvement and erosion prevention. As reviewed by Brooker et al. (2023), complementarity effects result from processes such as niche differentiation or plant facilitation. The weed trait database proposed by Barberi et al. (2018) could be further enriched and translated in a tailor-made way at the community level in order to take into account any interactions and limitations due to non-compatible, non-synchronized, or competitive species. Approaches relying on combinations of functional traits by means of multispecies ideotypes, as proposed by Gaba et al. (2015) and Lavorel and Garnier (2002), could also be applied to weed communities. Yvoz et al. (2021) identified that small weed species with a short life cycle and low competitiveness present the optimum proxy combination, i.e., high services and low harmfulness. It has to be noted also that the coexisting ability of the different species based on resource partitioning requires diverse traits and usually gives resistance to invasion by other species (Dukes, 2001; Gaba et al., 2015).

The management (manipulation) of weed communities in favor of weeds with desirable traits and the mitigation of any major disservices (Step 4) is crucial for the exploitation of weeds as ecosystem services’ providers. For instance, the conservation of natural vegetation and the manipulation of weed flora by means of mowing at different heights could be some good ideas for boosting specific species instead of others and enhancing ecosystem services. Additionally, the selection as cover crops or “service weeds” of some self-seeding species that provide adequate ground cover without being competitive with the main crops is also recommended (Carpio et al., 2020). In all cases and similar to service crops, even for weeds, we have to select the combinations that increase positive effects in the agroecosystems like pest deterrence and improved water infiltration (Rafflegeau et al., 2023) and mitigate crop competition and other disservices. Within this context, protocols and decision support systems focused on weeds, ecosystem services, and agroecological management could be developed (Kanatas et al., 2020).

Step 5, which refers to the evaluation of the performance of several practices and weed communities in the growing season, should not be underestimated since this will be the basis for any required adaptation and optimization of the overall strategy. Quantification of ecosystem services is necessary and can be achieved by means of field trials in a wide range of pedoclimatic conditions and multidisciplinary research combining diverse pros and cons (Rapidel et al., 2009; Gaba et al., 2015). Any assessment could shift from the single species to the community level. Moreover, when we talk about agricultural land, the effects of any practices and approaches on crop yields or exploitation of the produced biomass as forage should not be ignored (Daryanto et al., 2019; Robinson et al., 2022).

Regarding the obstacles, the major issue is to overcome the nudges regarding weeds. Indeed, farmers and agronomists are not used or trained to systematically recognize the ecosystem services provided by plants, particularly by spontaneous vegetation, and consequently to value and promote such benefits and communities. Thus, demonstration and training go first with an eye always to the local conditions, available labor and machinery, and top priorities. Training and technical support are crucial, especially during the first steps for the dispersal of the most beneficial species and the enhancement of the ecosystem services provided by the weed flora. Moreover, ecosystem services usually rely on a limited number of spontaneous or sown species. Therefore, further research is needed to study more species and their potential roles and interactions without risking the dominance of a few noxious and competitive species in the agroecosystem and the decrease in crop yields due to competition and allelopathy. The potential need for the termination of these “service weeds” should also be taken into account, exactly as it happens with cover crops.

## 3 Conclusions and perspectives

The strategic integration of non-crop plant diversity at field, farm, and landscape scales is an invaluable tool for achieving sustainability (Hawes et al., 2021). More diverse communities have a higher likelihood of some species functioning well under various conditions (“insurance hypothesis”), maintaining multiple ecosystem processes across multiple places and times, and contributing to more resilient agroecosystems (Isbell et al., 2011; Visconti et al., 2018). As Gaba et al. (2015) highlighted, determining plant diversity and designing and applying management practices that could deliver a set of targeted services under given environmental and socioeconomic conditions are crucial. The question of whether weeds can play a role or should be kept only as opponents (Travlos et al., 2023) is answered here: weeds do have a role to play in the agroecosystem. Due to their short life cycles, plasticity, and adaptability, the good traits that selection pressure gave them, the art of coexistence and complementarity that they have developed, and many other advantages, they can remarkably turn into “service weeds” and contribute to the already defined main ecosystem services. The obstacles related to their disservices, inadequate training, the difficult quantification of ecosystem services, and the nudges among farmers and other stakeholders can be overcome through multidisciplinary research, training, and policy initiatives. We propose the conservation, management, and optimization of a native weed flora (diversified) in perennial crops, field margins, strips, and between crop rows and its manipulation by means of mowing, grazing, deep tillage, etc., in favor of small growth and short growing cycle annual species towards a significant enhancement of ecosystem services. It is time to “unbox” the potential value of weeds for the agroecosystems, even if it is to redefine in our minds the term “weed”.

## Author contributions

IG: Conceptualization, Writing – review & editing, Writing – original draft. PK: Writing – review & editing, Writing – original draft. SZ: Writing – review & editing, Writing – original draft. MK: Writing – original draft. VK: Writing – original draft, Visualization. NA: Writing – original draft. IT: Writing – review & editing, Writing – original draft, Conceptualization.
